# Computational Ranking of Yerba Mate Small Molecules Based on Their Predicted Contribution to Antibacterial Activity against Methicillin-Resistant *Staphylococcus aureus*

**DOI:** 10.1371/journal.pone.0123925

**Published:** 2015-05-08

**Authors:** Caroline S. Rempe, Kellie P. Burris, Hannah L. Woo, Benjamin Goodrich, Denise Koessler Gosnell, Timothy J. Tschaplinski, C. Neal Stewart

**Affiliations:** 1 UT-ORNL Graduate School of Genome Science and Technology, University of Tennessee, Knoxville, TN, 37996, United States of America; 2 Department of Plant Sciences, University of Tennessee, Knoxville, TN, 37996, United States of America; 3 Department of Civil and Environmental Engineering, University of Tennessee, Knoxville, TN, 37996, United States of America; 4 Department of Electrical Engineering and Computer Science, University of Tennessee Knoxville, TN, 37996, United States of America; 5 Oak Ridge National Laboratory, Oak Ridge, TN, 37831, United States of America; ContraFect Corporation, UNITED STATES

## Abstract

The aqueous extract of yerba mate, a South American tea beverage made from *Ilex paraguariensis* leaves, has demonstrated bactericidal and inhibitory activity against bacterial pathogens, including methicillin-resistant *Staphylococcus aureus* (MRSA). The gas chromatography-mass spectrometry (GC-MS) analysis of two unique fractions of yerba mate aqueous extract revealed 8 identifiable small molecules in those fractions with antimicrobial activity. For a more comprehensive analysis, a data analysis pipeline was assembled to prioritize compounds for antimicrobial testing against both MRSA and methicillin-sensitive *S*. *aureus* using forty-two unique fractions of the tea extract that were generated in duplicate, assayed for activity, and analyzed with GC-MS. As validation of our automated analysis, we checked our predicted active compounds for activity in literature references and used authentic standards to test for antimicrobial activity. 3,4-dihydroxybenzaldehyde showed the most antibacterial activity against MRSA at low concentrations in our bioassays. In addition, quinic acid and quercetin were identified using random forests analysis and 5-hydroxy pipecolic acid was identified using linear discriminant analysis. We also generated a ranked list of unidentified compounds that may contribute to the antimicrobial activity of yerba mate against MRSA. Here we utilized GC-MS data to implement an automated analysis that resulted in a ranked list of compounds that likely contribute to the antimicrobial activity of aqueous yerba mate extract against MRSA.

## Introduction

Antibiotic resistance is a significant problem for human and animal health since the development and production of novel antimicrobials has lagged behind the evolution of bacteria for multi-drug resistance. Methicillin-resistant *Staphylococcus aureus* (MRSA) is a pathogenic biotype of global concern because of its resistance to multiple antibiotics [1]. Bioactive plant compounds are a potential source for novel antimicrobial formulations; however, the isolation and identification of a novel antimicrobial component among hundreds of compounds can be costly and time-consuming. Therefore, a prioritization of compounds based on bioactivity is a useful first step of an efficient antimicrobial discovery process.

Recent reports have shown that an aqueous extract of yerba mate tea, from the plant *Ilex paraguariensis*, is bactericidal and inhibitory to the growth of bacterial pathogens [2,3], including MRSA [4] and could contain one or more novel antimicrobial compounds. Although many compound classes could contribute to antimicrobial activity, this study focused on compounds, like phenolics, that are readily detected with gas chromatography-mass spectrometry (GC-MS) when derivitized with trimethylsilyl (TMS) groups. Small phenolic molecules are known to be prevalent in yerba mate [5] and have antimicrobial activity against many pathogens, including MRSA (reviewed in [6, 7]), and so serve as a reasonable initial focus in the search for yerba mate antimicrobial constituents. Phenolics previously observed in extracts of yerba mate tea include caffeic acid, caffeoylquinates, caffeoylshikimates, dicaffeoylquinates, feruloylquinates, kaempferol, quercetin, and rutin, which have been observed to have beneficial properties ranging from antioxidant to antitumor activities (reviewed in [5]). Recently, MRSA-antimicrobial activity of yerba mate was characterized [4, 8]. In addition, Martin et al. 2013 [8] identified chlorogenic acid and caffeic acid in the yerba mate extracts to be active against bacterial food pathogens, however, these compounds were not tested against MRSA.

The purpose of our study was to isolate and identify the compounds contributing to yerba mate’s antimicrobial activity against MRSA. This study expanded on previous assessments by both identifying compounds with antimicrobial activity against MRSA using a unique assembly of existing methods and testing authentic standards.

## Materials and Methods

### Yerba Mate Extractions

Dried leaves of a single commercial brand of yerba mate tea (Taragui; Argentina; 100% leaves; *I*. *paraguariensis*) were purchased from a local international supermarket. Extracts were obtained using previous methods [3] with modifications. Commercial tea leaves were finely ground to a particle size of less than 300 μm using a commercial food blender (Oster, Boca Raton, Fla., USA). Sterile deionized water was added to ground leaves at a ratio of 3.6 ml to 1 g ground tissue, was allowed to stand for 2 h at 4°C with occasional mixing to maximize extraction and was subsequently centrifuged at 5000 × g for 30 min. Aqueous extracts were then subjected to dialysis at 4°C against deionized water for 36 h using a 3500 MWCO SnakeSkin pleated dialysis tubing (ThermoFisher Scientific, Rockford, Ill., USA). Dialyzed extracts were then centrifuged at 5000 × g for 30 min to remove large insoluble particles and frozen at -80°C. Frozen extracts were lyophilized using Labconco FreeZone 12 L Freeze Dry System (Labconco, Kansas City, Missouri, USA) to concentrate them. Lyophilized extracts were stored at room temperature in a sealed container until testing.

The lyophilized aqueous extract was subsequently extracted with 10%, 20%, 30%, 40%, 50%, 60%, 70%, 80%, and 90% solvent (methanol or acetonitrile). Following solvent extraction, samples were centrifuged at 13,000 × g for 30 min which separated them into two fractions: the pellet (not soluble in solvent concentration) and the supernatant (soluble in solvent concentration). Fractions were subsequently dried using a SpeedVac Concentrator (Savant Industries, Inc., Farmingdale, N.Y., USA). Lyophilized solvent-extracts were weighed, resuspended in sterile water to a concentration of 40 mg/ml and stored at -20°C until testing. The GC-MS chromatograms of one initial active and one inactive methanol extract were used to select compounds found in the active extract but absent in the inactive extract, and likely to contribute to antibacterial activity (sugars were not selected).

### Antimicrobial Susceptibility Tests

MRSA strains ATCC 33591 and ATCC 33593 were purchased from American Type Culture Collection (ATCC; Manassas, Va., USA). Methicillin-sensitive *Staphylococcus aureus* (SA) strains ATCC 27708 and SA 113 were obtained from the Center Environmental Biotechnology at the University of Tennessee, Knoxville (courtesy of Steven Ripp). Bacteria were selected on Baird-Parker medium (Becton, Dickinson and Co., Sparks, Md., USA) and stock cultures were prepared by isolating a single colony, growing in tryptic soy broth (TSB; Becton, Dickinson and Co.) and stored at -20°C in glycerol.

Solvent extracts were tested for antimicrobial activity by the disk diffusion method against MRSA and non-resistant *S*. *aureus* (SA). Pure cultures of each bacterial strain were sub-cultured at least once in Mueller-Hinton broth (Becton Dickinson & Co.) by inoculating 50 ml broth with 200 μl stock cultures for 24 h incubation at 35–37°C. Following incubation, ca. 9.0 log_10_ CFU/ml cultures were diluted to ca. 6.0 log_10_ CFU/ml and each diluted bacterial suspension was swabbed onto Mueller-Hinton agar plates prior to disk placement. Twenty microliters of the extract or water control were placed on each 6 mm sterile blank disk (Becton Dickinson & Co.), and subsequently plated in duplicate on Mueller-Hinton agar. Plates were incubated for 24 h at 37°C and the zones of inhibition of bacteria were measured.

Two sets of activity assays were performed with authentic standards. The first used concentrations based on the approximated ratios of compounds observed in our initial two-sample GC-MS data (glycolic acid 1 μg/ml, 3,4-dihydroxybenzaldehyde 0.01 μg/ml, citric acid 31 μg/ml, caffeic acid 46 μg/ml, kaempferol 2 μg/ml, chlorogenic acid 285 μg/ml, 4-*O*-caffeoylquinic acid 210 μg/ml, 5-*O*-caffeoylquinic acid 311 μg/ml, and each of these concentrations at 10x). The second set of activity assays tested compounds alone at 10 μg/ml, 20 μg/ml, and 100 μg/ml. Activity assays for authentic standards alone and in combination were carried out using a micro-broth dilution assay. Sterile 96-well microtiter plates with a well capacity of 300 μl were used. A total volume of 250 μl was used consisting of 125 μl double strength tryptic soy broth (TSB), 10 μl chemical diluted in 1% dimethyl sulfoxide (DMSO) in water, and 25 μl of inoculum (ca. 6.0 log_10_ CFU/mL). For the second set of activity assays, samples were randomly assigned within central, intermediate, and edge locations on each plate to minimize edge effects. Only center samples were used in the final analysis. For all tests, microtiter plates were covered with a sterile lid and incubated for 24 h to 48 h at 37°C and the absorbance (630 nm) of each well was read at 0 h, 8 h, 24 h, and 48 h (for the second bioassay) with a microtiter plate spectrophotometer (El_x_800 Universal Microplate reader, BioTek Instruments, Winooski, Vermont, USA). The micro-broth dilution assays were performed in triplicate. Statistical analysis of the data was performed using analysis of variance with mixed models in SAS 9.4 (SAS Institute Inc., Cary, N.C., USA) using a randomized block design (RBD) blocked on replicate for each single strain separately at 48 h or 24 h. Least squares means were separated using Tukey’s significant difference test. Levene tests for equal variance (P>0.05) and Shapiro-Wilks tests for normality (W>0.80) were performed using R, and boxplots were constructed (R Foundation for Statistical Computing, Vienna, Austria).

### GC-MS Sample Preparation and Instrument Parameters

The samples rehydrated to 40 mg/ml were filtered using 0.2 μm nylon membrane filters (13 mm Acrodisc; Pall Corporation, Ann Arbor, Mich., USA). Fifty microliters of each sample (or the whole sample if less than 50 μl) and 15 μl of 1 mg/ml liquid sorbitol solution were then dried under a stream of sterile flowing nitrogen. Two samples that contained < 50 μl were multiplied by a correction factor after feature detection and retention time correction to approximate a 50 μl sample. Several samples contained insufficient material for rehydration, filtration, and derivitization. We analyzed a total of 60 samples by GC-MS, 34 of which were unique (some biological duplicates generated different amounts of material). Dried samples were derivitized by adding 500 μl HPLC grade acetonitrile followed by 500 μl N-methyl-N-trimethylsilyltrifluoroacetamide (MSTFA) with 1% trimethylchlorosilane (TMCS) (Thermo Fisher Scientific) and incubating at 70°C for 60 min. After 2 d, 1 μl of each sample was injected by an autosampler into a GC-MS instrument (Agilent Technologies Inc., Santa Clara, Calif., USA) with a 5975C inert XL gas chromatograph-mass spectrometer, fitted with an Rtx-5MS with Integra-guard (5% diphenyl/95% dimethyl polysiloxane) capillary column 30 m by 250 μm by 0.25 μm of film thickness). This standard quadrupole GC-MS was operated with electron impact (EI) ionization at 70 eV, six 50 Da to 650 Da scans per second, and helium gas flow rate of 1.33 ml/min with the injection port in splitless mode. Temperatures were held at 250°C for the injection port, 230°C for the mass spectrometer source, and 150°C for the mass spectrometer quad. The oven was programmed to start at 50°C for 2 min, ramp up to 325°C at 20°C per min, hold for 11 min, and then cycle back down to 50°C. Data files were exported to AIA format in MSD ChemStation (Agilent Technologies, Inc., Santa Clara, Calif, USA) for subsequent analysis.

### Data Analysis

In an initial assessment of methanolic fractions of yerba mate extract, we selected two comparable samples, one with antimicrobial activity against MRSA and one without antimicrobial activity against MRSA, for GC-MS analysis. The resulting spectra were overlayed and GC peaks that were higher in the active samples and lower in the inactive samples were identified with the aid of MS libraries when possible.

The software program XCMS [9] was used for both feature detection and retention time correction with parameters based on the “GC—EI, Single Quadrupole MS” default values from XCMSOnline [10,11]. The effectiveness of the feature detection of different parameter sets was assessed by plotting detected features onto a heatmap of a single sample with all known reference compounds present (S1 Fig). The presence of features at particular reference ions of known compounds was assessed alongside general trends of the heatmap topology in order to select optimal parameters. The same method was used to evaluate the effectiveness of correcting retention time drift by plotting corrected features and uncorrected features on the same single sample heatmap. Final feature detection and retention time correction parameters for XCMS are listed in S1 Table. Correction factors for the 2 samples with concentrations less than 50 μl were multiplied by intensity values to create a modified matrix that was used for all subsequent analyses. There was variation in the sorbitol (internal standard) 319 m/z peak height relative to the intensities of other peaks in some samples, which was likely the result of ion suppression and/or sample matrix interactions. Therefore, spectral peaks were further adjusted based on the 319 m/z peak of sorbitol, so that adjusted ion peak = ion peak * average sorbitol 319 m/z peak across all spectra/sorbitol 319 m/z peak of current spectrum.

Linear discriminant analysis (LDA) was implemented as a pseudo-inverse matrix calculation using the Python linear algebra library numpy.linalg.pinv. The data matrix from XCMS was normalized by subtracting the mean and dividing by the standard deviation for each attribute column. To construct a proper discriminant function, a 'bias' feature was added in the form of a column of ones. With this, the Python Numpy pseudo-inverse function was used to train and test the data with leave-one-out cross-validation sets. Finally, the whole dataset was used in training to generate an optimized weight for each attribute. The weight coefficient for each attribute in the discriminant function can be seen as an estimate of that attribute's importance in computing the antimicrobial activity. Hence, attributes were ranked from largest to smallest weight and assessed for the numbers of known antimicrobial compounds with high rank.

MetaboAnalyst was used to generate additional ranked peaklists using different methods. Using MetaboAnalyst software [12], we prioritized peaks in ranked lists from fold-change analysis (using proportions of change between two sample groups), t-test (using comparison of means between two groups), principal component analysis (PCA; using an unsupervised measure across data axis with the most spread), partial least squares linear discriminant analysis (PLS-DA; using a supervised measure across data axes with most variation), recursive support vector machine (R-SVM; using the supervised data-separating hyperplanes of several SVMs to rank the top 50 attributes from the t-test), significance analysis of microarray (SAM; using significance scores a thresholds to determine the significance of single features), empirical Bayesian analysis of microarray (EBAM; using a two group mixture model to separate null and significant features based on a delta value of 0.9), and random forests (using set of weak decision tree learners which form a strong voting system for classification) analyses. MetaboAnalyst parameters for no missing data and autoscaling for normalization were selected.

Lists from PCA were ordered from largest to smallest absolute values of the first principal component loading values to assess an unsupervised approach. PLS-DA, LDA, and random forests lists were ordered from largest to smallest (not absolute value) since they were supervised, and positive values were associated with attributes that discriminated the positive class (active) from the negative class (inactive). Lists from statistical tests were kept in order of statistical significance (smallest to largest p-values (t-test), smallest to largest log2s (fold change), largest to smallest z-values (EBAM), and largest to smallest d-values (SAM)). Ranking weights and statistical values are reported in S2 Table.

JMP Pro 10 (SAS Institute Inc., Cary, N.C., USA 1989–2007) was used to run logistic regression analyses on the top-ranked spectral peaks of quinic acid, quercetin, and 5-hydroxy-pipecolic acid using peak intensity as a continuous variable to predict a binary outcome of inactive or active against MRSA. Each model was evaluated based on the whole model test Prob<ChiSq p-value and lack of fit Prob<ChiSq p-value being less than 0.05. The parameter estimate of the model was then used to reverse predict the classes of the dataset based on the spectral peak to get a predictive accuracy.

Prioritized peak lists were compared in a pairwise fashion using a rank biased overlap function implemented with the Python function rbo.py [13,14]. This function scored the similarity between the single top-ranked element of each list, then scored the similarity between the top two-ranked elements of each list, and so on, in overlapping sections to generate an overall score. These scores gave greater weight to the more highly ranked elements by using a convergent series of weights, specifically the geometric series [13].

The Golm Metabolome Database (GMD) was used to predict functional groups for unknown compounds. Each functional group had its own decision tree classifier with a cross-validation accuracy obtained from the GMD [15] that was used to gauge predictive accuracy. The Python library urllib2 was used to interface with the GMD in an automated fashion.

Retention index was calculated from a best-fit line of known compounds based on their retention indices in GMD GC-EI-TMS spectra, and their retention times in our data. This calculation was devised from the knowledge that any set of compounds with known retention indices can be used to index other compounds from the same spectra [16]. Predicted functional groups were summarized for each dataset compilation by counting occurrences of each unique predicted group for all unique retention times and for the top ten unique retention times.

## Results

Yerba mate extracts fractionated by varying proportions of methanol (S3 Table) or acetonitrile (S4 Table) in water had variable activity against MRSA and SA. In general, fractions with higher antimicrobial activity were observed in supernatants extracted with 80% or less methanol and 70% or less acetonitrile and in the pellets remaining from the extractions using higher percent methanol (>80%) (S3 Table) or acetonitrile (>70%) fractions (S4 Table). The resulting activity data was used to label each fraction as ‘active’ (any measure of bacterial growth inhibition) or ‘inactive’ (no bacterial growth inhibition) (S3 and S4 Tables).

**Fig 1:**
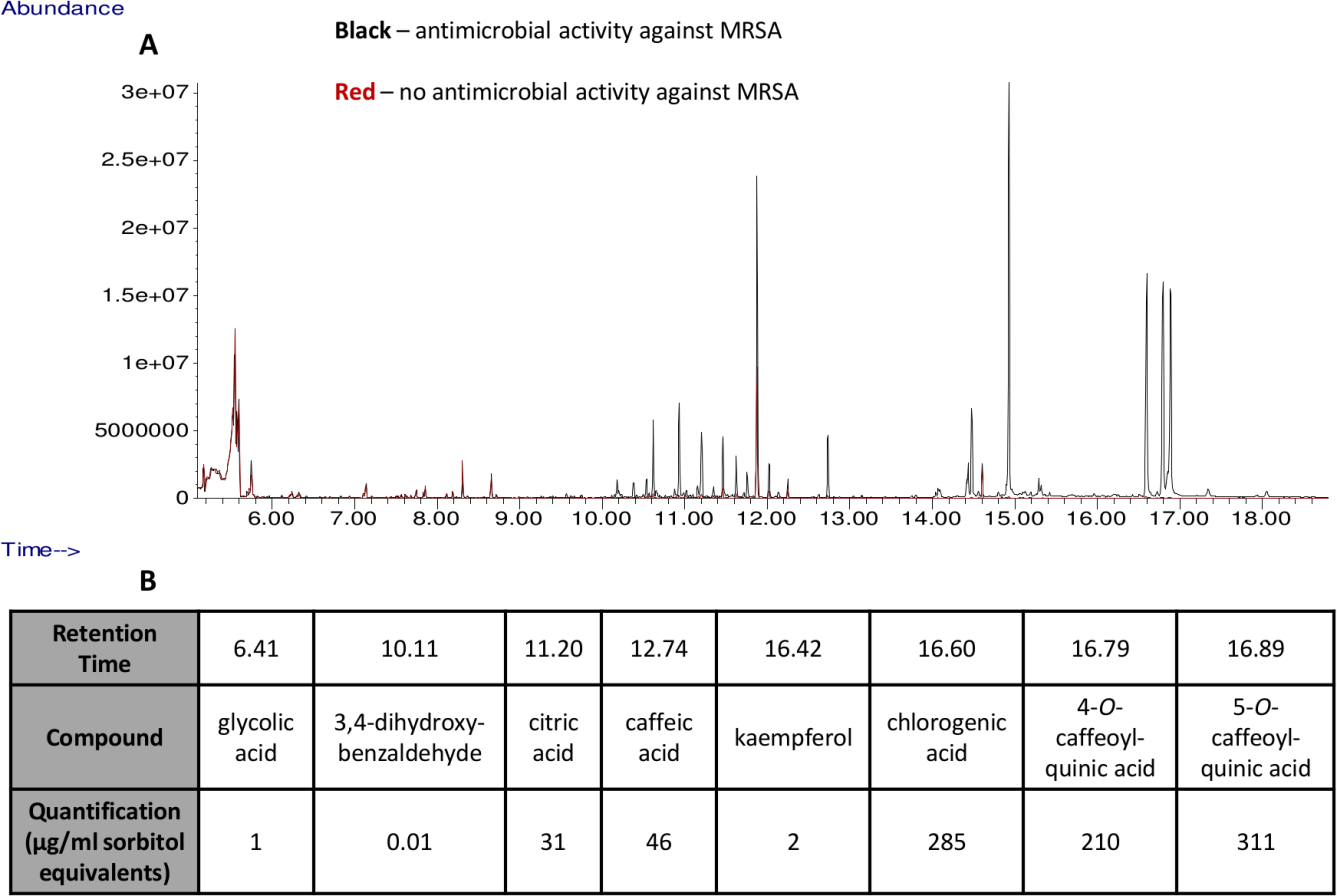
Overlay of initial yerba mate extract fraction chromatograms. A) The black chromatogram corresponds to a yerba mate extract fraction that demonstrated antibacterial activity against methicillin-resistant *Staphylococcus aureus* (MRSA); the red chromatogram corresponds to a yerba mate fraction that had no antibacterial activity against MRSA. B) Retention times of identified compounds and quantification in sorbitol equivalents were reported.

Our initial GC-MS analysis of one spectrum from an antibacterial extract and one spectrum from a non-antibacterial extract generated a differential list of 8 non-carbohydrate compounds (Fig 1), 4 of which were already known to inhibit MRSA, based on literature sources (S5 Table). For our automated spectral analysis of a more robust dataset, our feature detection parameters with XCMS resulted in 2204 m/z peaks at unique retention times. Our GC-MS data is available in the MetaboLights database, repository ID MTBLS170 (http://www.ebi.ac.uk/metabolights/MTBLS170). Visual examination of detected features on a m/z vs. retention time heatmap (S1 Fig) revealed that features generally aligned well with high intensity m/z regions of the heatmap. A check for the existence of major ions from our 8 known compounds showed that at least one major ion peak from caffeic acid, citric acid, and the chlorogenic acids was present. The extracted ion peaks our group used for 3,4-dihydroxybenzaldehyde and kaempferol were not detected, but other characteristic peaks of these compounds were observed at their respective retention times. Peaks for glycolic acid were not detected.

In a check for proper retention time correction, features from two different sampling time periods were visually observed to improve based on overlays before and after retention time correction plotted on a sample heatmap (S1 Fig). Although there was significant improvement, retention time drift correction could still be improved further, as evidenced by non-overlapping dots in the corrected plot.

In general, spectra from extracts of different solvent fractionation methods contained many of the same GC peaks, so differences in quantitative information were more relevant to this analysis than differences in qualitative information. The ability to rank influential compounds would be improved and perhaps more reliable with fractionation methods that generated extracts with very qualitatively different compositions. Nevertheless, our analysis of this dataset revealed an additional set of three identifiable, and potentially antibacterial, compounds (quinic acid, quercetin, and 5-hydroxy-pipecolic acid) in addition to the unknown compounds that ranked with known antimicrobials. Since the list ranked by random forests contained the most antimicrobial compounds that were known in high ranks, we additionally surveyed compounds at ranks between 10 and 20. We identified one unknown (rank 13) compound predicted to be aromatic in the 10–20 ranks of the random forests list. This unknown compound would be of interest to further characterize as a potential novel antimicrobial (Table 1). In our analysis of activity against just SA, 3*-O-*feruloylquinic acid was identified as an additional compound of interest.

**Table 1:** Top 20 unique retention times ranked by antimicrobial significance against MRSA using random forests.

Rank	Retention Time (min.)	Known Active Concentration (μg/ml)	Major Ion Peaks	Name
**1**	11.44	---	147, 255, 345	Quinic acid
**2**	11.18	900	147, 273	Citric acid
**3**	12.71	250	219, 396	Caffeic acid
**4**	14.92	---	147, 217, 361	Sucrose
**5**	16.41	10	396, 559	Kaempferol
**6**	16.82	125	307	Quercetin
**7**	10.8	---	394	Unknown
**8**	16.58	500	147, 255, 307, 345	3*-O-*caffeoylquinic acid
**9**	18.03	---	103, 129, 204, 217, 361, 427	Raffinose
**10**	5.61	---	144, 158	DA
**11**	10.58	---	55, 57, 69, 75, 81, 83, 97, 99, 123, 204, 217	Unknown
**12**	16.78	---	193, 255, 257, 324, 372, 489	4*-O-*caffeoylquinic acid
**13**	12.58	---	143	Unknown, predicted aromatic
**14**	6.23	---	295	DA
**15**	5.11	---	59, 86, 100, 133, 160, 174, 175, 221, 223	DA
**16**	14.47	---	-	Unknown
**17**	15.28	---	103, 129, 204, 217, 305, 361	Unknown
**18**	16.85	---	133, 191, 239, 283, 357, 419, 447	5*-O-*caffeoylquinic acid
**19**	10.07	---	262	Unknown
**20**	11.85	---	275	DA

Known active concentrations were obtained from either literature or our bioassays.

DA derivitization artifact

—no known inhibitory concentration found

- no peaks above cut-off

**Fig 2:**
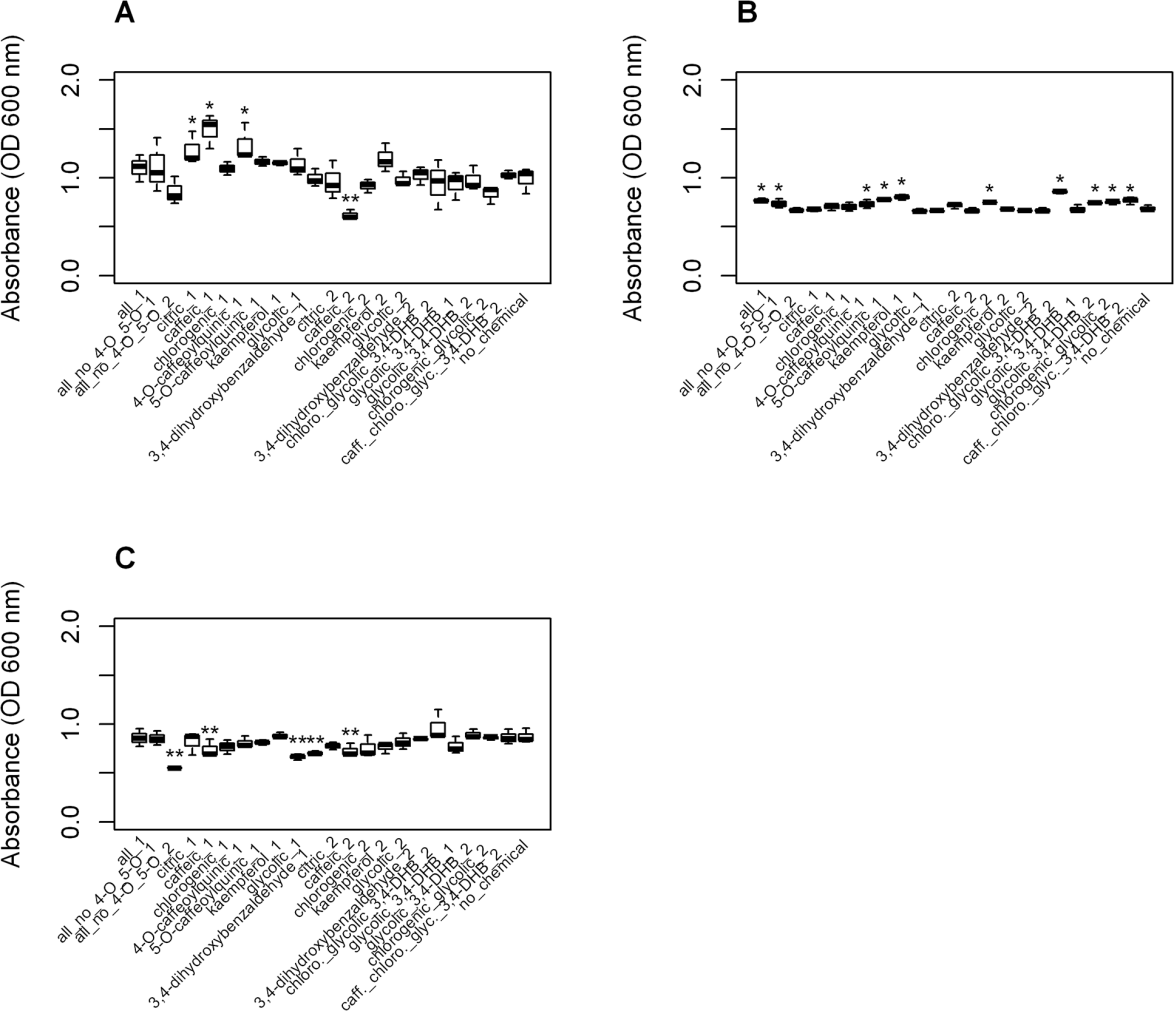
Growth of methicillin-sensitive (SA) and methicillin-resistant *Staphylococcus aureus* (MRSA) in the presence of single or multiple pure compounds at proportions approximated from GC-MS quantification. Growth with compounds alone or together was compared to the positive growth control (no chemical added) to determine inhibitory activity. Statistically significant differences greater (*) or less (**) than control are marked by asterisks. Concentrations follow the GC-MS quantification values in Fig 1, but in μg/ml. Growth of A. SA 27708, B. MRSA 35591, and C. MRSA 35593 are reported at 24 h.

We evaluated the following eight pure compounds that were identified in our initial GC-MS data overlay for antimicrobial activity alone and in selected combinations at concentrations based on GC-MS quantification (in sorbitol equivalents) to assess approximate antimicrobial ratios of the compounds: citric acid, caffeic acid, 3,4-dihydroxybenzaldehyde, 3*-O-*caffeoylquinic acid (chlorogenic acid), 4*-O-*caffeoylquinic acid, 5*-O-*caffeoylquinic acid, kaempferol, and glycolic acid. We observed a significant reduction (P<0.05) in means relative to a positive growth control sample (mean of 0.99 absorbance units, standard error of mean (SE) 0.08) with caffeic acid at 460 μg/ml (0.62 absorbance units, SE 0.08) with SA 27708. We also observed a significant reduction (P<0.05) in means relative to a positive growth control sample (0.87 absorbance units, SE 0.05) with MRSA 33593 for 3,4-dihydroxybenzaldehyde at 0.01 μg/ml (0.70 absorbance units, SE 0.05), glycolic acid at 1 μg/ml (0.66 absorbance units, SE 0.05), and caffeic acid at both 46 μg/ml (0.73 absorbance units, SE 0.05) and 460 μg/ml (0.72 absorbance units, SE 0.05). Of our selected combinations of compounds, only the sample containing the combination of most components (caffeic acid, citric acid, 3,4-dihydroxybenzaldehyde, chlorogenic acid, kaempferol, and glycolic acid at 10x concentrations listed in Fig 1) showed antimicrobial activity (0.55 absorbance units, SE 0.05) against MRSA, and only against MRSA 33593 (Fig 2).

**Fig 3:**
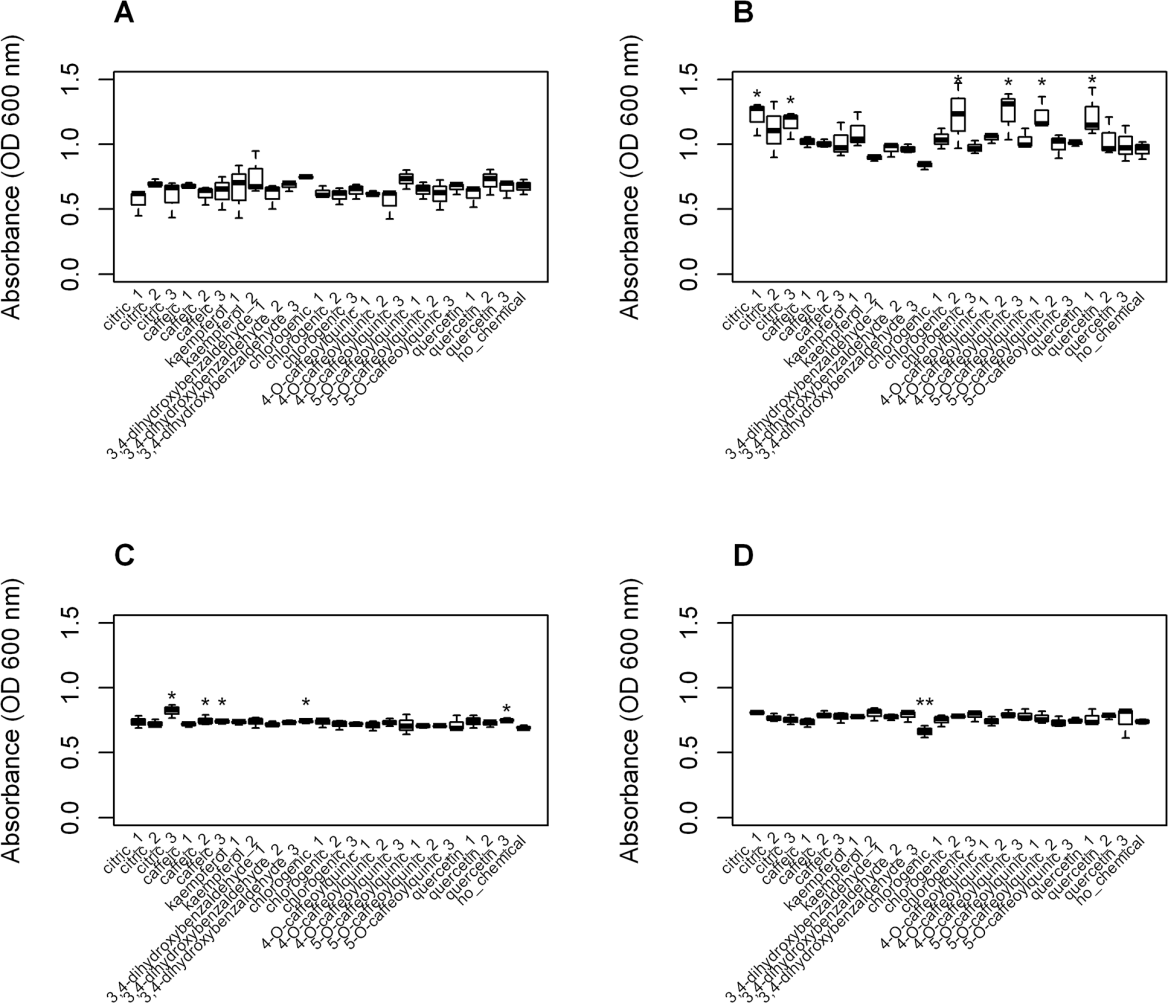
Growth of methicillin-sensitive (SA) and methicillin-resistant *Staphylococcus aureus* MRSA) in the presence of pure compounds. At concentrations of 10 μg/ml (chemical_1), 20 μg/ml (chemical_2) and 100 μg/ml (chemical_3), growth with compounds was compared to the positive growth control (no chemical added) to determine inhibitory activity. Statistically significant differences greater (*) or less (**) than control are marked by asterisks. Growth of A. SA 113, B. SA 27708, C. MRSA 35591, and D. MRSA 35593 are reported at 48 h. SA113 had a significant block by treatment interaction, so no conclusions can be drawn from it.

Each of the previously-identified compounds were also tested at concentrations of 10 μg/ml, 20 μg/ml, and 100 μg/ml, although only 3,4-dihydroxybenzaldehyde was observed to significantly reduce MRSA growth (from positive control mean of 0.74 absorbance units, SE 0.03, to 0.66 absorbance units, SE 0.03), and only at 100 μg/ml (Fig 3). Assumptions of normality, equal variance, and no block-by-treatment interaction were upheld with the exception of SA 113 (Fig 3A), in which a significant block-by-treatment interaction was observed (P < 0.05), which meant that no conclusion could be drawn from Fig 3A.

A total of 12 ranked peak lists were produced using statistical tests and classification analyses from MetaboAnalyst software and LDA. Among the lists generated by supervised learning (PLS-DA, random forests, LDA), the accuracies were similar, with an accuracy of 0.83 for both LDA and random forests with the MRSA data, and accuracies of 0.86 for SA LDA and 0.80 for SA RF. PLS-DA had an accuracy of 0.77 for both MRSA and SA (confusion matrices in S6 Table). There was no obvious similarity between misclassified samples, although several samples had relatively low intensities.

The relationship between result lists from different classification methods was assessed using a rank biased overlap analysis [13] and are summarized in Table 2. The lists generated by t-test, SAM, and EBAM were nearly identical (RBO > 0.99 similarity), so only the t-test list is displayed in Table 3. PCA, fold-change, and SVM lists were most different from each other and other lists (RBO < 0.1). The fold-change list was additionally noted to contain many derivitization artifacts with high rank (7 of the top 10 were derivitization artifacts). Random forests, PLS-DA, and t-test results were more similar to each other with RBO values ranging from 0.2 to 0.6 (RBO similarity scale 0 to 1, 1 = identical). Many of the same compounds appeared in the top 10 elements of each list, summarized in Table 3. These compounds included citric acid, 3*-O-*caffeoylquinic acid, 4*-O-*caffeoylquinic acid, caffeic acid, quinic acid, quercetin, and unknown compounds (retention times and major m/z peaks listed in Table 3).

**Table 2:** Rank biased overlap comparison of lists.

**Average RBO MRSA**									
	PCA	Fold Change	PLS-DA	SAM	SVM	T-test	LDA	EBAM	RF
PCA	1	3.08E-11	0.000366	0.000192	0.002276	0.001582	0.000109	0.001605	0.008998
Fold Change	3.08E-11	1	0.011483	0	0	7.34E-06	0.075296	1.23E-09	0.000891
PLS-DA	0.000366	0.011483	1	0.618936	0.05196	0.612391	0.012616	0.612037	0.136071
SAM	0.000192	0	0.618936	1	0.002872	0.999874	0.00313	1	0.224733
SVM	0.002276	0	0.05196	0.002872	1	0.004933	0.04423	0.004054	0.024031
T-test	0.001582	7.34E-06	0.612391	0.999874	0.004933	1	0.002991	0.999644	0.220768
LDA	0.000109	0.075296	0.012616	0.00313	0.04423	0.002991	1	0.002995	0.009479
EBAM	0.001605	1.23E-09	0.612037	1	0.004054	0.999644	0.002995	1	0.220807
RF	0.008998	0.000891	0.136071	0.224733	0.024031	0.220768	0.009479	0.220807	1
**Average RBO SA**									
	PCA	Fold Change	PLS-DA	SAM	SVM	T-test	LDA	EBAM	RF
PCA	1	1.59E-12	2.95E-05	1.13E-05	6.17E-09	0.000676	0.000333	0.00068	0.010393
Fold Change	1.59E-12	1	0.044871	0	0	3.28E-05	0.098322	1.86E-11	0.001522
PLS-DA	2.95E-05	0.044871	1	0.395548	0.28188	0.388904	0.081874	0.388895	0.149397
SAM	1.13E-05	0	0.395548	1	0.527674	1	0.015228	1	0.186309
SVM	6.17E-09	0	0.28188	0.527674	1	0.542135	0.051292	0.542135	0.122868
T-test	0.000676	3.28E-05	0.388904	1	0.542135	1	0.0154	0.999991	0.182801
LDA	0.000333	0.098322	0.081874	0.015228	0.051292	0.0154	1	0.015403	0.021609
EBAM	0.00068	1.86E-11	0.388895	1	0.542135	0.999991	0.015403	1	0.182803
RF	0.010393	0.001522	0.149397	0.186309	0.122868	0.182801	0.021609	0.182803	1

Values range from 0, dissimilar, to 1, identical. For comparison between MRSA and SA lists, rank biased overlap between MRSA RF and SA RF = 0.19; MRSA LDA and SA LDA = 0.057; PCA = 1; PLS-DA = 0.399; t-test = 0.518, EBAM = 0.518; SVM = 0; fold change = 0.63.

**Table 3:** Top ten results for each attribute ranking method.

Classification Method		1		2	3	4	5	6	7	8	9	10
**MRSA Linear Discriminant Analysis**	**Name or mz peaks**	DA		532, 398, 396	293	glucose	140, 214, 187, 124	236	5-hydroxy-pipecolic acid	_	185	palmitic acid
	**RT (min)**	5.11		16.25	12.1	11.98	9.15	14.83	9.94	14.47	9.38	12.22
**MRSA Random Forests**	**Name or mz peaks**	quinic acid		citric acid	caffeic acid	sucrose	kaempferol	quercetin	394, 511	3-*O*-caffeoylquinic acid	raffinose	DA
	**RT (min)**	11.44		11.18	12.71	14.92	16.41	16.82	10.8	16.58	18.03	5.61
**MRSA Recursive Support Vector Machine**	**Name or mz peaks**	211		228	sorbitol	DA	492	5-*O*-caffeoylquinic acid	314, 301, 299	5-hydroxy-pipecolic acid	246	626, 624, 166, 324
	**RT (min)**	8.33		14.96	11.85	11.89	13.82	16.85	7.84	9.94	11.13	17.3
**MRSA Partial Least Squares Discriminant Analysis**	**Name or mz peaks**	sorbitol		citric acid	211	DA	503	sucrose	211	333	133, 218, 103, 205, 117, 149, 101	quinic acid
	**RT (min)**	11.85		11.19	8.33	11.89	11.87	14.93	7.24	12.4	7.82	11.44
**MRSA T-test**	**Name or mz peaks**	sorbitol		citric acid	416	quinic acid	sucrose	3-*O*-caffeoylquinic acid	183	394, 511	355, 415, 429	_
	**RT (min)**	11.85		11.18	14.93	11.44	14.89	16.59	10.67	10.8	11.89	11.54
**MRSA Fold Change**	**Name or mz peaks**	DA		124	246	DA	DA	DA	DA	DA	DA	172
	**RT (min)**	6.31		14.18	12.04	6.02	7.53	5.89	5.34	5.75	6.1	9.58
**Principal Component Analysis**	**Name or mz peaks**	glycerol-3-phospate		168	174, 100, 184, 134, 227, 285, 86, 130, 77, 69, 59	3-*O*-caffeoylquinic acid	quercetin	5-*O*-caffeoylquinic acid	4-*O*-caffeoylquinic acid	sucrose	3-*O*-feruloylquinic acid	217, 219
	**RT (min)**	10.88		8.93	8.29	16.58	16.84	16.87	16.81	14.91	16.49	10.9
**SA Linear Discriminant Analysis**	**Name or mz peaks**	4-*O*-caffeoylquinic acid	DA		301, 168	368	3-*O*-caffeoylquinic acid	172	185, 258	5-*O*-caffeoylquinic acid	457, 247, 57, 512, 349, 483, 455, 263, 248, 207, 514	124, 187, 214, 140
	**RT (min)**	16.78	5.11		8.94	13.21	16.59	9.58	9.55	16.85	14.58	9.15
**SA Random Forests**	**Name or mz peaks**	citric acid	quinic acid		183, 416	337, 275, 375, 292, 377	sorbitol	288, 129, 306	217, 129, 103, 75	3-*O*-feruloylquinic acid	170	217, 204, 75
	**RT (min)**	11.18	11.43		10.67	13.79	11.85	10.51	10.35	16.49	10.32	10.59
**SA Recursive Support Vector Machine**	**Name or mz peaks**	quinic acid	511, 394		292	_	citric acid	375, 292	183	258	360	257
	**RT (min)**	11.44	10.8		9.71	11.54	11.19	13.79	10.67	9.55	10.57	10.18
**SA Partial Least Squares Discriminant Analysis**	**Name or mz peaks**	335, 265	sorbitol		223, 205, 175, 263, 265, 191	citric acid	183, 416	345, 204	416, 268	258, 185	437, 363, 361, 271, 217, 103, 129	329
	**RT (min)**	9.24	11.85		5.77	11.19	10.67	14.9	9.55	9.32	9.65	12.4
**SA T-test**	**Name or mz peaks**	citric acid	quinic acid		183, 416	394, 511	258, 185	292	_	sorbitol	375, 292, 377, 275, 337	306, 288, 129
	**RT (min)**	11.19	11.44		10.67	10.8	9.55	9.71	11.54	11.85	13.79	10.51
**SA Fold Change**	**Name or mz peaks**	DA	DA		DA	DA	DA	246	DA	DA	DA	DA
	**RT (min)**	5.15	6.02		7.53	5.89	6.31	12.04	5.34	6.44	6.1	8.94

MLKRanking methods included linear discriminant analysis, random forests, recursive support vector machine, partial least squares linear discriminant analysis, t-test, and fold-change with data from MRSA or SA. Since principal component analysis is unsupervised, MRSA or SA labels have no influence and so there is only one PCA results list. Results are summarized as unique retention times (RT) with major (greater than *max intensity peak*/10 in the single sample that had the greatest intensity, highest ranked m/z peak) m/z peaks (mz) or chemical name if it is known. Known antimicrobial compounds are marked by colored boxes. Lists for SAM, EBAM, and t-test were identical for the top ten components, so only the t-test list is reported.

DA derivitization artifact

- no peaks above cut-off

Contributions of the newly-identified potential antimicrobial compounds quinic acid, quercetin, and 5-hydroxy-pipecolic acid were further assessed with a logistic regression analysis of a single representative m/z peak for each. Using m/z peaks 537, 471, and 244 for quinic acid, quercetin, and 5-hydroxy-pipecolic acid, respectively, we evaluated the area under receiver operating characteristic (ROC) curves for the logistic regression analysis of each compound (Table 4). The area under the ROC curve followed the ranked order of the compounds with quinic acid at 0.86, quercetin at 0.78, and 5-hydroxy-pipecolic acid at 0.72. All of these values are > 0.50, and so there exists some predictive ability over random chance.

**Table 4:** Classification accuracy of a single major mz peak for each of the 3 identified compounds of interest.

Area under receiver operating characteristics curve		
	MRSA	SA
quinic acid mz 537	0.83	0.86
quercetin mz 471	0.78	0.78
5-hydroxy-pipecolic acid mz 244	0.72	0.68

Quinic acid, quercetin, and 5-hydroxy-pipecolic acid classification accuracies are reported as the area under the curve (AUC) of a receiver operating curve (ROC) for the logistic regression of each single m/z peak MRSA or SA. Accuracy ranges from 0 (no samples accurately classified) to 1 (all samples accurately classified).

Additional compounds that could not be identified might also contribute to antimicrobial activity. To gain further information about the highly-ranked unknown compounds, functional groups were predicted by GMD decision trees for the fragments detected at each unique retention time in the random forests list (S7 Table and Table 5). For the 315 unique retention times found in our dataset, the major predicted functional groups were carboxylic acid derivatives (228), alpha amino acids (106), aromatics (119), and carboxylic acids (292) (Table 4). Of particular interest was the unknown compound in the random forests top-10 list, but the GMD decision trees predicted it to be a carboxylic acid and a carboxylic acid derivative rather than a functional group commonly associated with antibacterial activity. In examining the top-20 list from random forests, we found an unknown compound (rank 13) that was predicted to contain an aromatic group that would be of interest for further characterization as a potentially novel antimicrobial compound (Table 1).

**Table 5:** Predicted Functional Groups from Golm.

Unique Retention Time Counts from	MRSA Top 10	SA Top 10	All Retention Times
Carboxylic Acid Deriv	4	6	228
Alkene	0	0	3
Prim Aliph Amine	0	0	3
Alcohol	4	5	18
Alpha Aminoacid	0	0	106
Carbonyl	0	0	1
Prim Amine	0	0	4
Aromatic	5	3	119
Prim Alcohol	1	2	9
Phenol	2	0	3
1 2 Diol	3	2	9
Sec Alcohol	2	3	9
Phosphoric Acid Deriv	0	0	1
Carboxylic Acid	7	7	292
Hydroxy	2	0	10
Acetal	1	0	3
Amine	0	0	3

Summary of predicted functional groups from only the top 10 unique retention times and from all 315 unique retention times of the random forests ranked list.

The reliability of these functional group predictions was based on the “VAR5” cross-validation assessment of each training model done by the Golm group [15], with the resulting functional group predictions having an F-measure threshold of at least 0.65 based on a precision-recall plot [15]. The predicted functional group cross-validation error was 13.54% for “hydroxy“, 9.65% for “aromatic”, and 3.31% for “phenol” groups [15].

## Discussion

Hyphenated chromatography-mass spectrometry techniques have yielded a number of downstream data analysis pipelines (reviewed in [17]), from which we implemented functions from XCMS, MetaboAnalyst, and Python’s Numpy. We obtained GC-MS data for 60 fractions of aqueous yerba mate extract (including biological duplicates) and monitored the data-processing steps of XCMS. Since each fraction had a known bioactivity against MRSA, we computationally predicted compounds with significant contribution to the grouping, or class, of ‘active’ fractions and then assessed the classification using MICs found in the literature, as well as our own set of bioassays using authentic standards.

In our first bioassay, we aimed to assess the effect of key compounds alone and in combination using concentrations that approximated the relative content of compounds that were visible using GC-MS. We observed caffeic acid to be inhibitory at 46 μg/ml, which is similar to the 62.5 μg/ml previously observed for caffeic acid against SA, but smaller than the minimum inhibitory concentration (MIC) of 240 μg/ml reported against MRSA [18]. Citric acid and chlorogenic acid were previously shown to have MICs of 900 μg/ml [19] and 500 μg/ml or higher [18,20,21] for MRSA, respectively, although we did not observe these compounds to inhibit MRSA. We did observe the inhibition of MRSA by glycolic acid at 1 μg/ml, which is in the activity range of bacterial antibiotics [22]. However, glycolic acid was not found with our peak detection parameters, and so was not included in our peak ranking analysis or second bioassay. Kaempferol and quercetin were previously observed to be active against MRSA at pharmacologically-relevant concentrations of 13 μg/ml [23] and from 10 μg/ml to 125 μg/ml [20,24], respectively. However, we observed no significant growth inhibition for kaempferol or quercetin against MRSA at these concentrations (Fig 3). This lack of inhibition might result from the incomplete solubilization in 1% DMSO and, thus, loss of chemicals during filtration.

A major challenge of analyzing GC-MS data is dealing with the large number of attributes relative to the small number of samples. Data with more attributes than samples are easily overfit by nonlinear methods, so linear analysis methods are preferred. Since the purpose of the automated analysis was to rank attributes by their contribution to antimicrobial activity, we required a method capable of classifying and ranking samples. After a pre-processing step of feature detection and correction using XCMS software, we found that LDA was a method that fit both of these requirements, which has already been implemented in studies of mass spectral analysis [25,26]. MetaboAnalyst software also hosts a set of easily implemented techniques for the comparative analysis of mass spectral data that resulted in useful comparisons with LDA. This work suggested that LDA did not do well at prioritizing antimicrobial compounds with our data since none of the known antimicrobial compounds were ranked in the top-10 list (Table 3). The limitations of LDA with highly collinear data could have contributed to its poor rankings, as suggested previously [27]. Random forests classification implemented with MetaboAnalyst did, however, prioritize antimicrobial compounds well, ranking 4 of the known antimicrobials in the top-10 list. The superiority of random forests over SVM and LDA in a GC-MS classification application was previously observed [28]. Despite this apparent success of MetaboAnalyst’s random forests implementation, it also identified sugars in the top-10 list. This occurred in other lists too, as well as did derivization artifacts, or peaks that were present in the derivitization blank.

The highly-ranked matches to sugar compounds that we observed with multiple methods likely resulted from a coincidental correlation of sugar concentration with the yerba mate fractions observed to have antimicrobial activity. There are background sugar peaks at many unique retention times that could be glycosides attached to the active components we have identified, but the data generated did not give enough information to categorize them as glycosides or background sugar peaks. It is interesting to note that the presence of sugar has been observed to promote the activity of antimicrobial compounds [29].

Of the potential anti-MRSA compounds identified by our analysis that we were unable to experimentally test, 5-hydroxy-pipecolic acid and 3*-O-*feruloylquinic acid have strong potential for antimicrobial activity as hydroxylated phenolic compounds, even though we did not find any literature in which these compounds have been tested against MRSA. Quinic acid is a common plant metabolite in the shikimic acid pathway that has been reported to have antimicrobial activity against SA at 16 μg/ml, but it was not observed to have activity against MRSA at concentrations up to 28 μg/ml [30].

In the top-10 unique retention times predicted by random forests, there were an additional two aromatic, phenol, and hydroxyl groups predicted in the MRSA data over the SA data (Table 5). This identification suggests that aromatic and hydroxylated groups may play a role in the MRSA antimicrobial activity of the active fraction, although further analysis would be required to test this hypothesis. Aromatic compounds have antimicrobial activity dependant on hydroxyl groups (reviewed in [6]), including derivatives of caffeic acid and caffeoylquinics, which have previously been observed to inhibit MRSA [18].

## Conclusion

The increasing resistance of MRSA to existing antimicrobials demands the development of new antimicrobial options. Here we have assembled a pipeline that took advantage of the large amounts of data generated by GC-MS by implementing existing GC-MS tools for an automated analysis that resulted in a ranked list of compounds likely to contribute to the antimicrobial activity of aqueous yerba mate extract against MRSA. We tested the results of this analysis by assaying the antimicrobial activity of pure compounds at a pharmacologically-relevant concentration. 3,4-dihydroxybenzaldehyde was the only compound we assayed with activity at a concentration of 100 μg/ml or less. We also determined that 5-hydroxy-pipecolic acid, quercetin, quinic acid, and one unidentified compound could be possible contributors to yerba mate antimicrobial activity against MRSA using LDA and a random forests analysis. The unique combination of existing methods and tools used in this study generated prioritized lists of compounds likely to contribute to the antimicrobial activity of yerba mate extract, of which citric acid, caffeic acid, chlorogenic acid, kaempferol, quercetin were observed to inhibit MRSA in previous studies [18–20,23,31,32]. 5-hydroxy pipecolic acid, 3*-O-*feruloylquinic acid, and the unknown compound ranked 13 from the random forests list would be useful for further characterization in order to understand which natural compounds in yerba mate might serve as useful antimicrobials against both MRSA and SA.

## Supporting Information

S1 Fig Heatmaps showing feature detection and retention time correction of GC-MS data. A and B correspond to methicillin-resistant *Staphylococcus aureus* (MRSA) and C and D correspond to methicillin-sensitive *S*. *aureus* (SA). Blue and red dots are from samples obtained at different times that are in need of retention time correction. The A and C heatmaps display data points before retention time correction and the B and D heatmaps display data points after retention time correction. The complete overlap of blue over red would show perfect retention time correction.
(TIF)

S1 Table Final parameters implemented with XCMS for feature detection and retention time correction of mass spectral data. (DOCX)

S2 Table Ranking values of top 10 attributes for each ranking method. (DOC)

S3 Table Antimicrobial activity assays of aqueous yerba mate acetonitrile fractions. (DOCX)

S4 Table Antimicrobial activity assays of aqueous yerba mate methanol fractions. (DOCX)

S5 Table Summary of compounds identified as potential antibacterials from GC-MS data and MIC concentrations against methicillin-sensitive *Staphylococcus aureus* (SA) and methicillin-resistant *S*. *aureus* (MRSA) from literature. No information was found for inhibitory activity of 5*-O-*caffeoylquinic, 4*-O-*caffeoylquinic, 3,4-dihydroxybenzaldehyde, or 5-hydroxy-pipecolic acid against SA or MRSA in the literature.
(DOCX)

S6 Table Accuracies of classification methods shown in confusion matrices. The upper left corresponds to true negatives and the lower right corresponds to true positives.
(DOCX)

S7 Table Predicted functional groups listed for the top 10 compounds with retention time (RT), retention index (RI) and name if known. The name of the predicted functional group and its cross validation error rate (based on a GMD decision tree classifier for each functional group) is listed for each unique retention time.
(DOCX)
