# Supplementary figures and images for: Computational Ranking of Yerba Mate Small Molecules Based on Their Predicted Contribution to Antibacterial Activity against Methicillin-Resistant Staphylococcus aureus
Source: PLoS One. 2015 May 8;10(5):e0123925. doi: 10.1371/journal.pone.0123925 (PMC4425481; doi:10.1371/journal.pone.0123925)

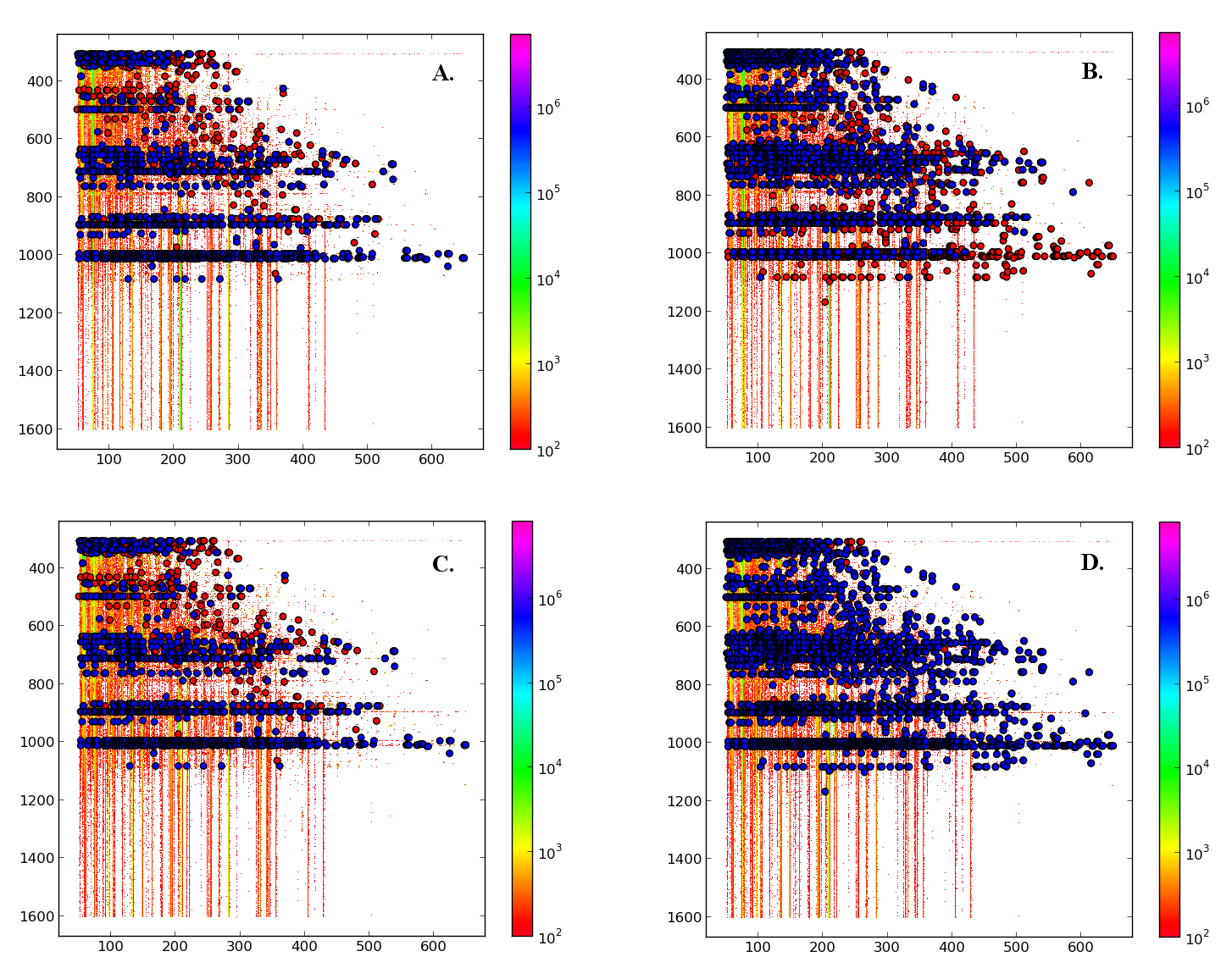

Supplement: S1 Fig — A and B correspond to methicillin-resistant Staphylococcus aureus (MRSA) and C and D correspond to methicillin-sensitive S. aureus (SA). Blue and red dots are from samples obtained at different times that are in need of retention time correction. The A and C heatmaps display data points before retention time correction and the B and D heatmaps display data points after retention time correction. The complete overlap of blue over red would show perfect retention time correction. (TIF) [file pone.0123925.s001.tif]
