# Supplementary material for: Computational Ranking of Yerba Mate Small Molecules Based on Their Predicted Contribution to Antibacterial Activity against Methicillin-Resistant Staphylococcus aureus
Source: PLoS One. 2015 May 8;10(5):e0123925. doi: 10.1371/journal.pone.0123925 (PMC4425481; doi:10.1371/journal.pone.0123925)
Supplement: S1 Table — (DOCX) [file pone.0123925.s002.docx]

**S1 Table**. **Final parameters implemented with XCMS** **for feature detection and retention time correction of mass spectral data.**

| **Feature Detection** | | |
| --- | --- | --- |
| **XCMS Parameters** | **Value** | **Function** |
| method | matchedFilter | Feature detection method using a matched filter algorithm that compares two classes of spectra to identify features |
| snthresh | 10 | Signal to noise threshold |
| max | 100 | Max peaks selected per mass chromatogram |
| step | 1.0 | Step size between masses (increased to match the unit mz resolution of our instrument) |
| bin | 2 | Steps per bin |
| mzdiff | 1 | Minimum difference in mz for peaks with overlapping retention times |
| **Retention Time Correction** | | |
| **XCMS Parameters** | **Value** | **Function** |
| method | peakgroups | “well behaved” peak groups are used to calculate retention time deviation, which is used for alignment |
| missing | 2 | Allowed missing peakgroups |
| extra | 2 | Allowed extra peakgroups |
| smoothing | loess | Loess smoothing method implemented |
| family | Gaussian | Check for Gaussian fit and symmetry; outlier check |
| span | 0.20 | degree of smoothing for regression fitting |
| **group()** Function to group peaks with below parameters | | |
| mzwid | 1 | Width of overlapping mz slices |
| minfrac | 1 | Minimum fraction of samples for peak group to be in, in at least one sample group |
| minsamp | 1 | Minimum number of samples for peak group to be in |
| bw | 5 before rtcorr,  3 after rtcorr | Bandwidth of Gaussian smoothing kernel |
