# Supplementary material for: Computational Ranking of Yerba Mate Small Molecules Based on Their Predicted Contribution to Antibacterial Activity against Methicillin-Resistant Staphylococcus aureus
Source: PLoS One. 2015 May 8;10(5):e0123925. doi: 10.1371/journal.pone.0123925 (PMC4425481; doi:10.1371/journal.pone.0123925)
Supplement: S2 Table — (DOC) [file pone.0123925.s003.doc]

**S2 Table. Ranking values of top 10 attributes for each ranking method.**

| **MRSA** | | | | | | **SA** | | | | | | |
| --- | --- | --- | --- | --- | --- | --- | --- | --- | --- | --- | --- | --- |
|  | **LDA** | **RF** | **SVM MST** | **PLS-DA MST** | **PCA MST** | **T-test MST** | **LDA** | **RF** | **SVM MST** | **PLS-DA MST** | **PCA MST** | **T-test MST** |
| **Ranking Scheme** | weight | Mean Decrease Accuracy | Frequency | Overall coefficient | abs(PC1 loading ) | p.value | weight | Mean Decrease Accuracy | Frequency | Overall | abs(PC1 loading) | p.value |
| **1** | 0.090635 | 0.003116 | 58 | 100 | 8.63E-06 | 2.24E-07 | 0.177646 | 0.002213 | 60 | 100 | 8.63E-06 | 3.14E-08 |
| **2** | 0.059662 | 0.002675 | 57 | 77.474 | 4.24E-05 | 1.65E-06 | 0.101643 | 0.002147 | 60 | 92.548 | 4.24E-05 | 4.19E-08 |
| **3** | 0.059054 | 0.00233 | 56 | 77.308 | 1.34E-06 | 1.75E-06 | 0.091323 | 0.001954 | 60 | 91.225 | 1.34E-06 | 1.09E-07 |
| **4** | 0.054956 | 0.002304 | 55 | 75.846 | 0.030877 | 2.08E-06 | 0.076261 | 0.001902 | 60 | 90.64 | 0.030877 | 1.36E-07 |
| **5** | 0.05391 | 0.002055 | 55 | 73.566 | 0.030661 | 2.26E-06 | 0.072665 | 0.001685 | 60 | 87.783 | 0.030661 | 1.38E-07 |
| **6** | 0.04643 | 0.001967 | 54 | 73.087 | 0.030577 | 2.67E-06 | 0.070089 | 0.001553 | 60 | 87.579 | 0.030577 | 1.47E-07 |
| **7** | 0.045362 | 0.001949 | 54 | 72.167 | 0.030535 | 3.08E-06 | 0.06767 | 0.001501 | 60 | 86.684 | 0.030535 | 1.50E-07 |
| **8** | 0.043524 | 0.001868 | 54 | 70.632 | 0.030486 | 3.24E-06 | 0.064844 | 0.0014 | 60 | 84.471 | 0.030486 | 1.53E-07 |
| **9** | 0.041844 | 0.001857 | 54 | 70.463 | 0.030448 | 3.51E-06 | 0.062407 | 0.001358 | 60 | 80.483 | 0.030448 | 1.92E-07 |
| **10** | 0.041506 | 0.001816 | 54 | 69.268 | 0.030409 | 3.53E-06 | 0.06196 | 0.001332 | 60 | 79.828 | 0.030409 | 2.09E-07 |
