# Supplementary material for: Computational Ranking of Yerba Mate Small Molecules Based on Their Predicted Contribution to Antibacterial Activity against Methicillin-Resistant Staphylococcus aureus
Source: PLoS One. 2015 May 8;10(5):e0123925. doi: 10.1371/journal.pone.0123925 (PMC4425481; doi:10.1371/journal.pone.0123925)
Supplement: S3 Table — (DOCX) [file pone.0123925.s004.docx]

**S3 Table**. **Antimicrobial activity assays** **of aqueous yerba mate acetonitrile fractions**.

| **Sample number** | **Sample type**  **(Supernatant)** | **Activity** | | | |
| --- | --- | --- | --- | --- | --- |
|  |  | **SA 27708** | | **MRSA 33591** | |
|  |  | **A** | **B** | **A** | **B** |
| 1 | Water super | Active | Active | Active | Active |
| 2 | 100% MeOH super | Active | None | Active | Active |
| 3 | 90% MeOH super | Active | None | None | Active |
| 4 | 80% MeOH super | Active | None | Active | Active |
| 5 | 70% MeOH super | Active | Active | Active | Active |
| 6 | 60% MeOH super | Active | Active | Active | Active |
| 7 | 50% MeOH super | Active | Active | Active | Active |
| 8 | 40% MeOH super | Active | Active | Active | Active |
| 9 | 30% MeOH super | Active | Active | Active | Active |
| 10 | 20% MeOH super | Active | None | Active | Active |
| 11* | Water pellet | None | None | None | None |
| 12 | 100% MeOH pellet | Active | Active | Active | Active |
| 13 | 90% MeOH pellet | Active | None | Active | Active |
| 14 | 80% MeOH pellet | Active | None | None | Active |
| 15 | 70% MeOH pellet | None | None | None | None |
| 16 | 60% MeOH pellet | None | None | None | None |
| 17 | 50% MeOH pellet | Active | Active | None | None |
| 18 | 40% MeOH pellet | None | None | None | None |
| 19 | 30% MeOH pellet | Active | None | None | None |
| 20 | 20% MeOH pellet | Active | Active | None | None |

Shaded boxes indicate that not enough extract was obtained to perform bioassays.

A and B are replicates.

Any size zone of inhibition was considered active.
