# Supplementary material for: Computational Ranking of Yerba Mate Small Molecules Based on Their Predicted Contribution to Antibacterial Activity against Methicillin-Resistant Staphylococcus aureus
Source: PLoS One. 2015 May 8;10(5):e0123925. doi: 10.1371/journal.pone.0123925 (PMC4425481; doi:10.1371/journal.pone.0123925)
Supplement: S4 Table — (DOCX) [file pone.0123925.s005.docx]

**S4 Table. Antimicrobial activity assays** **of aqueous yerba mate methanol fractions**.

| **Sample number** | **Sample type**  **(Supernatant)** | **Activity** | | | |
| --- | --- | --- | --- | --- | --- |
|  |  | **SA 27708** | | **MRSA 33591** | |
|  |  | **A** | **B** | **A** | **B** |
| 1 | Water super | Active | Active | Active | Active |
| 2 | 10% Acetonitrile super | Active | Active | Active | Active |
| 3 | 20% Acetonitrile super | Active | Active | Active | Active |
| 4 | 30% Acetonitrile super | Active | Active | Active | Active |
| 5 | 40% Acetonitrile super | Active | Active | Active | Active |
| 6 | 50% Acetonitrile super | Active | Active | Active | Active |
| 7 | 60% Acetonitrile super | Active | Active | Active | Active |
| 8 | 70% Acetonitrile super | None | Active | Active | Active |
| 9 | 80% Acetonitrile super | None | None | None | None |
| 10 | 90% Acetonitrile super | None | None | None | None |
| 11* | 100% Acetonitrile super | None |  |  |  |
| 12 | Water pellet |  |  |  |  |
| 13* | 10% Acetonitrile pellet | None | None |  |  |
| 14 | 20% Acetonitrile pellet | None | None |  |  |
| 15 | 30% Acetonitrile pellet | None | None | None | None |
| 16 | 40% Acetonitrile pellet | None | None | None | None |
| 17 | 50% Acetonitrile pellet | None | None | None | None |
| 18 | 60% Acetonitrile pellet | None | None | None | None |
| 19 | 70% Acetonitrile pellet | None | None | Active | None |
| 20 | 80% Acetonitrile pellet | Active | Active | Active | Active |
| 21 | 90% Acetonitrile pellet | Active | Active | Active | Active |
| 22 | 100% Acetonitrile pellet | Active | Active | None | None |

Shaded boxes indicate that not enough extract was obtained to perform bioassays.

A and B are replicates.

Any size zone of inhibition was considered active.
