# Supplementary material for: Computational Ranking of Yerba Mate Small Molecules Based on Their Predicted Contribution to Antibacterial Activity against Methicillin-Resistant Staphylococcus aureus
Source: PLoS One. 2015 May 8;10(5):e0123925. doi: 10.1371/journal.pone.0123925 (PMC4425481; doi:10.1371/journal.pone.0123925)
Supplement: S5 Table — No information was found for inhibitory activity of 5-O-caffeoylquinic, 4-O-caffeoylquinic, 3,4-dihydroxybenzaldehyde, or 5-hydroxy-pipecolic acid against SA or MRSA in the literature. (DOCX) [file pone.0123925.s006.docx]

**S5 Table.** **Summary of compounds identified as potential antibacterials from GC-MS data and MIC concentrations against methicillin-sensitive *Staphylococcus aureus* (SA) and methicillin-resistant *S. aureus* (MRSA) from literature.**

| **Compound** | **SA MIC** | **MRSA MIC** | **References** |
| --- | --- | --- | --- |
| Caffeic acid | 62.5 ug/ml | 250 ug/ml | LuÍs et al. 2013 |
| Citric acid | not found | 900 ug/ml;  0.05-0.2 gm% | Nagoba et al. 1998; Thool et al. 2014 |
| Chlorogenic acid | 200 ug/ml;  500 ug/ml | 500 ug/ml | Zhu et al. 2004;  LuÍs et al. 2013 |
| Kaempferol | not found | 10 ug/ml;  MIC_50_ = 13 ug/ml | Fattouch et al. 2007;  Hazni et al. 2008 |
| Quercetin | 10 ug/ml | 50uM;  125 ug/ml | Hirai et al. 2010;  Su et al. 2014 |
| Quinic acid | 16 ug/ml | >28 ug/ml | Özçelik et al. 2011 |
