# Supplementary material for: Computational Ranking of Yerba Mate Small Molecules Based on Their Predicted Contribution to Antibacterial Activity against Methicillin-Resistant Staphylococcus aureus
Source: PLoS One. 2015 May 8;10(5):e0123925. doi: 10.1371/journal.pone.0123925 (PMC4425481; doi:10.1371/journal.pone.0123925)
Supplement: S6 Table — The upper left corresponds to true negatives and the lower right corresponds to true positives. (DOCX) [file pone.0123925.s007.docx]

**S6 Table.** **Accuracies** **of classification methods shown in confusion matrices.**

|  | **MRSA** |  | |  |  |  | | |  | **SA** | |  | | |  | |  | |  |
| --- | --- | --- | --- | --- | --- | --- | --- | --- | --- | --- | --- | --- | --- | --- | --- | --- | --- | --- | --- |
|  | **LDA**  **Predicted** | |  | **RF**  **Predicted** | |  |  |  | | | **LDA**  **Predicted** | | |  | | **RF**  **Predicted** | |  | |
|  | **-1 1** |  | | **-1** | **1** |  | | |  | **-1 1** | |  | | | **-1** | | **1** | |  |
| **-1** | 11 4 | **-1** | | 9 | 6 |  | | | **-1** | 8 10 | | **-1** | | | 11 | | 7 | |  |
| **1** | 6 39 | **1** | | 4 | 41 |  | | | **1** | 5 37 | | **1** | | | 5 | | 37 | |  |
|  |  |  | |  |  |  | | |  |  | |  | | |  | |  | |  |
|  | **PLS-DA accuracy** | | |  |  |  | | |  | **PLS-DA accuracy** | | | | |  | |  | |  |
|  | 0.77 |  | |  |  |  | | |  | 0.77 | | |  | |  | |  | |  |
