# Supplementary material for: Computational Ranking of Yerba Mate Small Molecules Based on Their Predicted Contribution to Antibacterial Activity against Methicillin-Resistant Staphylococcus aureus
Source: PLoS One. 2015 May 8;10(5):e0123925. doi: 10.1371/journal.pone.0123925 (PMC4425481; doi:10.1371/journal.pone.0123925)
Supplement: S7 Table — The name of the predicted functional group and its cross validation error rate (based on a GMD decision tree classifier for each functional group) is listed for each unique retention time. (DOCX) [file pone.0123925.s008.docx]

**S7 Table.** **Predicted functional groups listed for the top 10 compounds with retention time (RT), retention index (RI) and name if known.**

| **Rank** | **RT** | **RI** | **Name** | **Predicted Functional Groups** |
| --- | --- | --- | --- | --- |
| 1. **MRSA** | | | | |
| 1 | 11.43 | 1983.451 | quinic acid | Carboxylic Acid, Alcohol,  Carboxylic Acid Deriv., Aromatic |
| 2 | 11.18 | 1930.634 | citric acid | Sec Alcohol,  Carboxylic Acid,  Alcohol,  Carboxylic Acid Deriv. |
| 3 | 12.72 | 2254.577 | caffeic acid | Hydroxy,  Aromatic,  Carboxylic Acid, Phenol, Carboxylic Acid Deriv. |
| 4 | 14.92 | 2719.366 | sucrose | None |
| 5 | 16.42 | 3036.268 | kaempferol | Carboxylic Acid, Aromatic |
| 6 | 16.82 | 3120.775 | quercetin | 1 2 Diol,  Carboxylic Acid, Aromatic |
| 7 | 10.8 | 1849.648 | unknown | Carboxylic Acid, Carboxylic Acid Deriv. |
| 8 | 16.58 | 3071.479 | 3-O-caffeoylquinic | Carboxylic Acid, Hydroxy Aromatic,  1 2 Diol,  Phenol Alcohol |
| 9 | 18.03 | 3377.817 | raffinose | 1 2 Diol  Sec Alcohol,  Acetal,  Alcohol,  Prim Alcohol |
| 10 | 5.62 | 754.5775 | derivitization artifact | none |
| 1. **SA** | | | | |
| 1 | 11.18 | 1930.634 | citric acid | Sec Alcohol,  Carboxylic Acid,  Alcohol,  Carboxylic Acid Deriv. |
| 2 | 11.43 | 1983.451 | quinic acid | Carboxylic Acid, Alcohol,  Carboxylic Acid Deriv., Aromatic |
| 3 | 10.67 | 1821.479 | unknown | Carboxylic Acid, Carboxylic Acid Deriv. |
| 4 | 13.78 | 2479.93 | unknown | Carboxylic Acid, Carboxylic Acid Deriv.,  Aromatic |
| 5 | 11.85 | 2071.479 | derivitization artifact | Carboxylic Acid, Aromatic |
| 6 | 10.52 | 1789.789 | unknown | Carboxylic Acid, Alcohol,  Carboxylic Acid Deriv |
| 7 | 10.35 | 1754.577 | unknown | 1 2 Diol  Sec Alcohol,  Alcohol,  Prim Alcohol, |
| 8 | 16.5 | 3053.873 | 3-O-caffeoylquinic | Carboxylic Acid, Aromatic, |
| 9 | 10.32 | 1747.535 | unknown | Carboxylic Acid, Carboxylic Acid Deriv. |
| 10 | 10.58 | 1803.873 | unknown | Sec Alcohol,  Alcohol,  1 2 Diol  Prim Alcohol |
